# Nursing-led multidisciplinary ERAS collaboration improves early recovery after laparoscopic radical prostatectomy for localized prostate cancer: a retrospective cohort study

**DOI:** 10.3389/fmed.2025.1705709

**Published:** 2026-01-14

**Authors:** Chunmei Chen, Yumei Li, Xueyan Chen, Ping Zhang, Shuang Zhu

**Affiliations:** 1 Disinfection and Supply Department, The Affiliated Traditional Chinese Medicine Hospital, Southwest Medical University, Luzhou, Sichuan, China; 2 Operating Room, The Affiliated Traditional Chinese Medicine Hospital, Southwest Medical University, Luzhou, Sichuan, China

**Keywords:** patient care team, prostatic neoplasms, perioperative care, urinary incontinence, prostatectomy

## Abstract

**Background:**

Postoperative recovery after laparoscopic radical prostatectomy (LRP) is influenced by perioperative nursing care. Therefore, this retrospective study aimed to compare outcomes from nursing-led perioperative multidisciplinary nursing collaboration based on Enhanced Recovery After Surgery (PMNC) with standard care in 235 patients undergoing LRP for localized prostate cancer.

**Methods:**

A retrospective cohort study was conducted in 235 patients with localized prostate cancer undergoing LRP at a tertiary hospital in Southwest China. Patients received either standard care (*n* = 115, routine perioperative nursing in 2020–2021) or PMNC (*n* = 120, implemented in 2022), which comprised eight structured ERAS-based interventions delivered jointly by nurses, anesthesiologists, and urologists. Primary outcomes were time to ambulation, catheter removal, urinary incontinence at discharge, and hospital stay; secondary outcomes included pain at 24/48 h, complications, 30-day readmission, and time to oral intake. Outcomes were compared using *t*-tests, Mann–Whitney U, chi-square, or Fisher’s exact tests, with significance set at *P* < 0.05.

**Results:**

The PMNC group showed significantly earlier ambulation (14.1 vs. 17.8 h, *P* < 0.001), earlier catheter removal (4.4 vs. 5.4 days, *P* < 0.001), shorter hospital stay (5.2 vs. 6.1 days, *P* < 0.001), and lower incontinence at discharge (32.5% vs. 56.5%, *P* < 0.001). Pain scores were also lower at 24 h (*P* < 0.001) and 48 h (*P* < 0.001). Multivariate logistic regression confirmed PMNC as an independent protective factor for early continence (OR = 0.47, 95% CI: 0.28–0.80, *P* = 0.005).

**Conclusion:**

A structured PMNC model was associated with improved early recovery after LRP, including faster mobilization, earlier catheter removal, lower incontinence rates, shorter hospitalization, and reduced pain.

## Introduction

Prostate cancer is one of the most common malignancies affecting men worldwide, with a steadily increasing incidence in aging populations (1). For patients with localized disease, laparoscopic radical prostatectomy (LRP) remains a standard treatment modality offering favorable oncological control and minimally invasive advantages such as reduced bleeding and shorter recovery time (2, 3). Recent comparative research between robot-assisted and standard laparoscopic radical prostatectomy has reported differences in estimated blood loss and postoperative pain, highlighting that procedural refinements alone may not fully address perioperative recovery challenges (4). However, functional outcomes—particularly urinary continence and early mobilization—continue to pose major challenges following LRP, often leading to prolonged hospital stays, patient dissatisfaction, and increased healthcare costs (5).

In recent years, enhanced perioperative care models have gained traction as a means to improve surgical recovery (6). Notably, Enhanced Recovery After Surgery (ERAS) programs have demonstrated benefits in colorectal and gynecologic surgery by optimizing preoperative education, pain control, mobilization, and complication prevention (6, 7). The ERAS approach is based on evidence-based perioperative protocols designed to reduce surgical stress, maintain physiological function, and accelerate postoperative recovery. Core elements typically include structured preoperative counseling, minimization of fasting, multimodal analgesia, early ambulation, and early resumption of diet, all supported through multidisciplinary collaboration (8). Within the broader ERAS framework, we define perioperative multidisciplinary nursing collaboration (PMNC) as a nursing-led multidisciplinary ERAS collaboration—that is, a team-based operationalization of key ERAS elements delivered jointly by ward and operating-room nurses in coordination with urologists and anesthesiologists. In this manuscript, PMNC is used synonymously with a multidisciplinary collaborative nursing approach embedded in an ERAS pathway, rather than a separate concept. By integrating structured education, standardized communication, coordinated intraoperative support, and early functional rehabilitation, PMNC translates ERAS concepts into daily clinical practice with a focus on continuity of care and interprofessional synergy. Despite these advances, urologic surgeries—especially LRP—have not fully adopted such models, and evidence specific to prostate cancer remains limited (9).

Furthermore, although nurse-led survivorship or psychoeducational programs for men with prostate cancer have begun to emerge, rigorous evaluations of nurse-led, multidisciplinary perioperative recovery models—particularly around radical prostatectomy—remain scarce (10). Nurses, serving as the cornerstone of perioperative care, are well-positioned to implement structured, evidence-based interventions across the continuum of surgical recovery (11). A multidisciplinary nursing collaboration model (PMNC), characterized by coordinated actions among nurses, anesthesiologists, and surgical teams, may offer a pragmatic and scalable strategy to enhance early recovery outcomes (12).

To address this gap, we developed and implemented a PMNC protocol composed of eight standardized, nurse-led components spanning the preoperative to postoperative phases. Therefore, this retrospective cohort study was designed to compare perioperative recovery outcomes between nursing-led multidisciplinary collaborative ERAS nursing (PMNC) and standard perioperative care in 235 patients undergoing laparoscopic radical prostatectomy for localized prostate cancer. We hypothesized that a structured, team-based nursing model would result in superior functional outcomes compared to conventional perioperative care. These findings may inform the development of pragmatic, nursing-driven recovery pathways in urologic oncology.

## Materials and methods

### Study design and setting

This study was conducted in accordance with the Declaration of Helsinki and approved by the Ethics Committee of The Affiliated Traditional Chinese Medicine Hospital, Southwest Medical University (Approval Number: BY2025050). The need for individual informed consent was waived due to the retrospective design and anonymization of data. This retrospective cohort study was conducted at the Department of Urology and Perioperative Nursing of a tertiary hospital in Southwest China. The overall study period spanned from January 2020 to December 2023 and encompassed two consecutive patient cohort s based on the implementation timeline of a standardized perioperative multidisciplinary nursing collaboration (PMNC) protocol. Patients treated from January 2020 to December 2021, prior to PMNC adoption, comprised the historical control group receiving conventional perioperative care. Patients treated between January 2022 and December 2023, following full protocol implementation, formed the PMNC intervention group. The primary objective was to evaluate the impact of PMNC on postoperative recovery outcomes in patients undergoing laparoscopic radical prostatectomy (LRP) for localized prostate cancer.

### Participants

**FIGURE 1:**
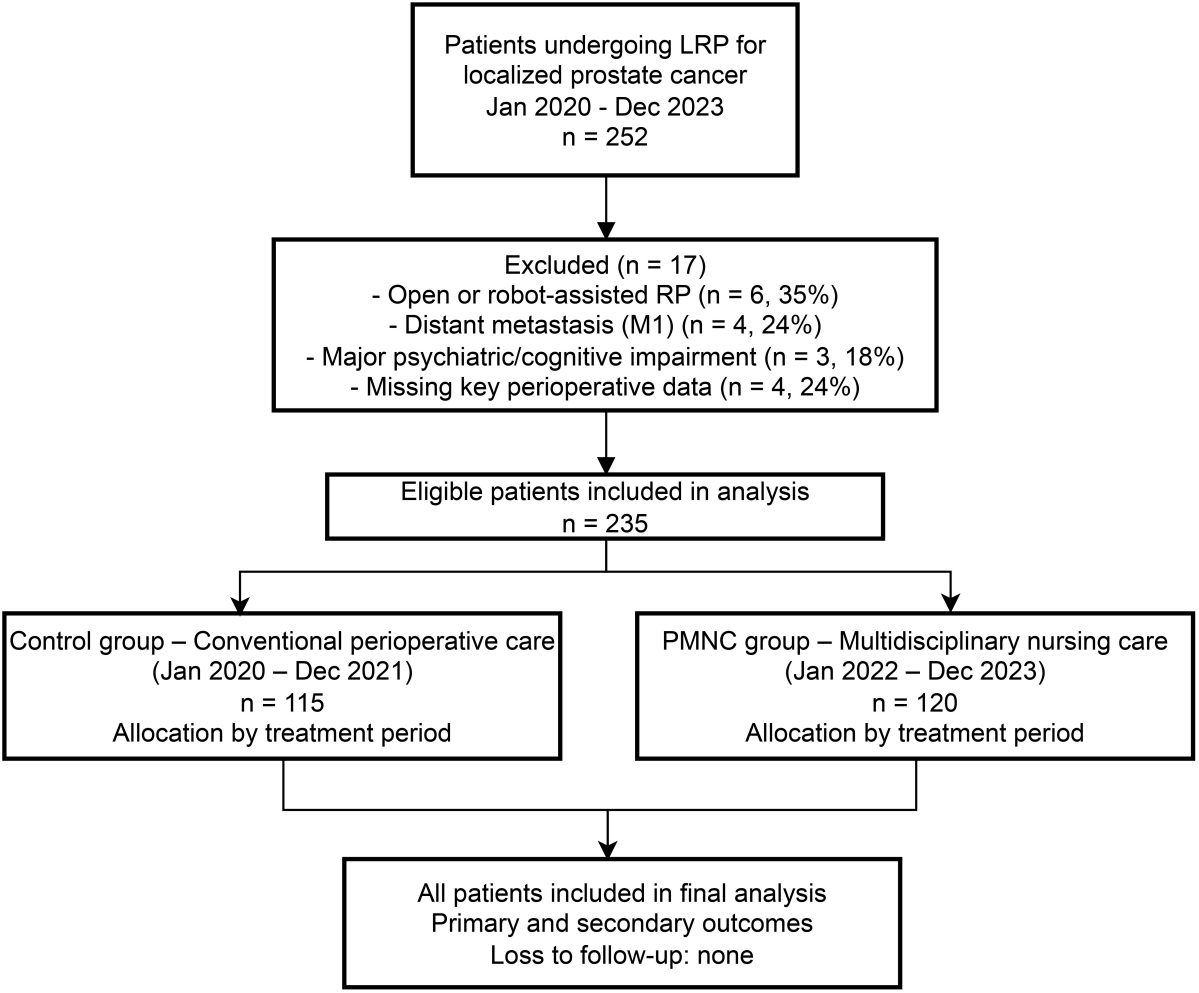
Patient selection and group allocation flowchart.

Eligible participants were male patients aged ≥ 50 years with histologically confirmed localized prostate cancer (clinical stages T1–T3, N0–N1, M0), who underwent LRP and had complete electronic medical and perioperative nursing records available. Exclusion criteria included patients who received open or robot-assisted prostatectomy, presented with distant metastasis (M1), had major psychiatric or cognitive impairments limiting perioperative cooperation, or had missing documentation related to perioperative care. A detailed patient screening and allocation process is illustrated in Figure 1, which depicts the number of patients assessed for eligibility, reasons for exclusion, and final group allocation. All included patients had localized prostate cancer (clinical stages T1–T3, N0–N1, M0). The distribution of clinical stage and Gleason grade was similar between groups, with no significant differences, as later detailed in the Results. Baseline demographic variables, including age, body mass index, ASA physical status, and comorbidities such as hypertension and diabetes, were collected to ensure comparability between groups. No patients received neoadjuvant hormonal therapy, chemotherapy, or radiotherapy prior to surgery, and there were no differences in non-surgical treatments between groups.

### Group assignment

Patients were assigned to either the PMNC or control group based on the time period in which they underwent surgery. Those treated between January 2022 and December 2023, during the hospital-wide implementation of the perioperative multidisciplinary nursing collaboration (PMNC) protocol, comprised the intervention group. Patients who received surgery between January 2020 and December 2021—prior to the introduction of PMNC—formed the control group. During the control period, no elements of the PMNC protocol were applied, either partially or selectively. All surgeries were performed by the same urologic team, and group assignment was retrospectively determined through the hospital’s electronic health record system. The lead surgeons remained consistent across both periods, with no changes in the primary operating surgeons or core intraoperative nursing staff, ensuring stability of surgical technique and intraoperative management practices throughout the study period. The core laparoscopic radical prostatectomy (LRP) technique, including patient positioning, trocar placement, dissection planes, and vesicourethral anastomosis approach, remained unchanged between the two study periods. Rates of nerve-sparing procedures, suture materials and methods, and extent of pelvic lymph node dissection were stable across both groups, as documented in operative reports. All procedures were performed using a transperitoneal laparoscopic approach under general anesthesia. Vesicourethral anastomosis was performed with a continuous running suture in the majority of cases (> 75%). Where nerve-sparing was indicated, an interfascial technique was applied. No cases were performed using a pure extraperitoneal or robot-assisted approach during the study period. Because the surgical technique and extent of lymphadenectomy were consistent across both cohorts, outcomes were not analyzed separately by approach. For all patients, laparoscopic radical prostatectomy was performed with the patient in the lithotomy position under general anesthesia. A standard five-port transperitoneal approach was used, including careful dissection of the prostate and seminal vesicles and vesicourethral anastomosis with a running suture. Nursing requirements during this procedure included strict verification of patient positioning to avoid nerve or pressure injury, monitoring of intraoperative fluid balance and thermoregulation, ensuring secure fixation of catheters and drainage tubes, and postoperative surveillance for bleeding, urinary leakage, and early mobilization readiness. These perioperative nursing requirements were consistent across both groups and provided the foundation upon which the PMNC interventions were added.

### Anesthesia and analgesia management

All patients received general anesthesia maintained with sevoflurane and remifentanil infusions titrated to hemodynamic stability. No epidural, neuraxial, or peripheral nerve blocks were used during the study period. Intraoperative analgesia included intravenous fentanyl and remifentanil, supplemented by acetaminophen (1 g) and flurbiprofen axetil (50 mg) administered before wound closure. Postoperative analgesia consisted of patient-controlled intravenous analgesia (PCIA) containing sufentanil (2 μg/mL; basal rate 2 mL/h; bolus 0.5 mL; lockout interval 15 min). Non-opioid adjuncts (acetaminophen 1 g every 8 h or flurbiprofen 50 mg every 12 h) were given if the visual analogue scale (VAS) score was ≥ 4, with reassessment within 1 h. Total postoperative opioid use was low and comparable between groups; therefore, morphine-equivalent conversion was not performed.

### Intervention: nursing-led multidisciplinary ERAS collaboration (PMNC)

In this study, PMNC refers to a nursing-led multidisciplinary ERAS collaboration, i.e., the translation of ERAS principles into protocolized, nurse-delivered actions across the perioperative period. The multidisciplinary approach consisted of coordinated contributions from ward nurses (education, mobilization, pain assessment), operating-room nurses (SBAR handover, positioning, thermoregulation and fluid monitoring), anesthesiologists (intraoperative hemodynamic and analgesia management), and urologists (surgical planning and participation in multidisciplinary rounds), thereby ensuring that each ERAS element was delivered through structured teamwork. The PMNC team comprised ward nurses (primary/charge), operating-room scrub and circulating nurses, urologists (attending/residents), and anesthesiologists; allied staff (e.g., rehabilitation or psychology) participated as available. Ward nurses were primarily responsible for patient education, psychological support, mobilization, continence training, and pain assessment; operating-room nurses coordinated SBAR handover, patient positioning, and intraoperative thermoregulation/fluid monitoring; anesthesiologists ensured hemodynamic stability and optimized perioperative analgesia; urologists supervised surgical planning and contributed to multidisciplinary rounds; and allied staff supported rehabilitation and psychological counseling when indicated. The PMNC model comprised eight standardized, nurse-led components collaboratively executed across the perioperative period: (1) preoperative education; (2) psychological support; (3) Situation–Background–Assessment–Recommendation (SBAR)-based handover between ward and operating room nurses; (4) intraoperative positioning checklist execution; (5) thermoregulation and fluid management monitoring; (6) early mobilization within 24 h postoperatively; (7) Pain was assessed using a 0–10 Visual Analog Scale (VAS) at 24 and 48 h postoperatively. Analgesia was adjusted if VAS ≥ 4, with reassessment within 1 h. The VAS was selected because it is the standardized pain assessment tool adopted by our institution for all surgical inpatients, including adults, and has been widely validated for use in coherent adult populations undergoing postoperative recovery. Its visual continuum allows patients to indicate pain intensity precisely, which facilitates consistent documentation and comparison across perioperative settings. (8) Structured multidisciplinary nursing rounds on postoperative day. All components were guided by predefined operating procedures, with documentation audited via nursing records. A side-by-side comparison of PMNC versus conventional perioperative care is provided in Supplementary Table 1 to clarify the additional elements introduced by the PMNC protocol.

### Standard care (control group)

In contrast, the control group received routine perioperative care without protocolized education, standardized handover tools, or collaborative recovery assessments. Conventional perioperative care during the control period (January 2020–December 2021) followed the standard nursing practices of our institution at that time. Preoperative preparation typically consisted of brief verbal instructions regarding fasting, medication use, and admission logistics, without structured educational materials or formalized teach-back confirmation. Psychological assessment was not routinely performed unless requested by the physician. Intraoperative coordination between ward and operating room staff occurred via informal verbal handover without a standardized SBAR format. Patient positioning was conducted according to surgeon preference, and formal checklists were not mandated. Thermoregulation and fluid monitoring were based on intraoperative nursing judgment without predefined targets. Postoperative mobilization was initiated at the discretion of the primary nurse, often delayed until the first postoperative day, and pain assessments were conducted only on patient request rather than at fixed intervals. Multidisciplinary nursing rounds were not standard practice, with care planning primarily led by the attending surgeon and individual nurses. A side-by-side comparison of PMNC versus conventional perioperative care is provided in Supplementary Table 1 to clarify the additional elements introduced by the PMNC protocol.

The PMNC model was a structured, nurse-led approach implemented collaboratively across the perioperative period, integrating eight coordinated elements from preoperative preparation to postoperative recovery. In the preoperative phase, trained perioperative nurses conducted a 15–20 min face-to-face education session using illustrated handouts, covering the surgical procedure, postoperative mobilization goals, pelvic floor muscle training, pain management expectations, and catheter care, with patient understanding confirmed via teach-back. Psychological support was provided 1 day before surgery through brief anxiety screening (Hospital Anxiety and Depression Scale) and targeted counseling on coping strategies and relaxation breathing. During ward-to-operating room transfers, a standardized SBAR (Situation–Background–Assessment–Recommendation) handover tool was used: the “Situation” section included patient identity, planned procedure, and surgical site; “Background” covered diagnosis, comorbidities, allergies, and laboratory results; “Assessment” summarized vital signs, IV lines, drains, and skin condition; and “Recommendation” outlined intraoperative considerations and postoperative care plans. Intraoperatively, a positioning checklist was jointly completed by scrub and circulating nurses to ensure correct lithotomy position, protect pressure points, and secure lines and catheters. Core temperature was maintained at ≥ 36.0°C using active warming devices, and fluid balance was monitored hourly. Postoperatively, patients were assisted to sit within 8 h and walk ≥ 10 m within 24 h unless medically contraindicated. Pain was assessed using a 0–10 Visual Analog Scale (VAS) at 24 and 48 h, with analgesia adjusted if VAS ≥ 4 and reassessed within 1 h. On postoperative day 1, a structured multidisciplinary nursing round involving the ward nurse, charge nurse, and surgical resident was conducted to review pain control, mobility, continence, wound care, and discharge planning. In contrast, the control group received routine perioperative care, which consisted of unstructured preoperative instructions, informal handovers without SBAR, variable intraoperative positioning checks, no formal temperature or fluid monitoring protocol, mobilization as tolerated without specific targets, pain assessment only as needed without fixed time points, and ward rounds conducted by individual nurses without a multidisciplinary format.

### Outcomes and ERAS evaluation

Enhanced recovery after surgery (ERAS) was evaluated using predefined, objectively measurable outcomes. Primary outcomes included time to first ambulation (hours from PACU arrival to walking ≥ 10 m), time to urinary catheter removal (days), urinary incontinence at discharge (≥ 1 pad/day documented by nursing records), and length of hospital stay (days). These variables reflect core ERAS goals of early mobilization, timely catheter removal, and shortened hospitalization. Secondary outcomes included postoperative pain scores at 24 and 48 h using a 0–10 Visual Analog Scale (VAS), Clavien–Dindo grade ≥ II complications, 30-day readmission, and time to oral intake resumption (hours to first tolerated liquid diet). These represent ERAS targets for effective pain control, complication prevention, and early functional recovery. Process evaluation of ERAS implementation was performed by calculating adherence rates to each PMNC component (see Table 4), which were audited weekly by a quality assurance nurse manager. Together, these outcome and process indicators provided a comprehensive assessment of ERAS effectiveness.

### Data collection

Data were extracted from electronic medical and nursing records using a structured abstraction form by two independent reviewers. Objective outcome definitions were applied consistently: time to first ambulation was defined as the interval from end of surgery to the first documented mobilization > 10 m; time to urinary catheter removal was the number of days until catheter removal confirmed in nursing records; urinary incontinence at discharge was defined as ≥ 1 pad/day, as recorded in standardized nursing continence assessments; length of hospital stay was calculated from admission to official discharge order. Pain was evaluated using the institutional 0–10 visual analog scale (VAS) administered at 24 and 48 h postoperatively by trained nursing staff. Postoperative complications were classified using the Clavien–Dindo system (grade ≥ II), and 30-day readmission was identified from hospital electronic records. Adherence to PMNC components was assessed by audit of mandatory nursing documentation fields. Two independent reviewers abstracted all variables using the standardized form; any discrepancies were resolved by consensus with a senior reviewer, while blinding to group assignment was maintained throughout abstraction. Missing data were assessed using Little’s MCAR test; variables with sporadic missingness (< 5%) were handled by complete-case analysis without imputation, and cases with > 10% missingness in critical variables were excluded as prespecified.

### Sample size calculation

Based on an expected medium effect size (Cohen’s *d* = 0.5) in time to ambulation between groups, with a significance level of 0.05 and power of 80%, the estimated minimum sample size was 64 per group. To ensure sufficient statistical power and account for potential exclusions, we included at least 80 patients in each cohort.

### Statistical analysis

All analyses were conducted using SPSS version 26.0 (IBM Corp., Armonk, NY, United States). Continuous variables were tested for normality using the Shapiro–Wilk test. Normally distributed variables were expressed as mean ± standard deviation (SD) and compared using independent-samples *t*-tests, while non-normally distributed variables were expressed as median with interquartile range (IQR) and compared using the Mann–Whitney U test. Categorical variables were expressed as counts and percentages and compared using chi-square or Fisher’s exact tests as appropriate. A multivariate logistic regression model was constructed to assess independent predictors of urinary incontinence, adjusting for age, BMI, ASA, and surgical factors. Odds ratios (ORs) with 95% confidence intervals (CIs) were reported. Model diagnostics included assessment of multicollinearity using variance inflation factors (VIFs) and overall goodness-of-fit using the Hosmer–Lemeshow test. Linearity in the logit for continuous covariates was examined and model specifications were retained if assumptions were satisfied. Pre-specified sensitivity analyses were performed to test robustness of findings: (i) excluding the first 6 months after PMNC rollout to mitigate transitional effects; (ii) including operative year as an additional covariate; and (iii) additionally adjusting models for operative duration and estimated blood loss. The direction and significance of the main effects were compared across these specifications. All statistical tests were two-sided, and a *P*-value < 0.05 was considered statistically significant.

### Bias control and data quality assurance

To minimize selection and measurement bias, all data abstractors were blinded to group assignment. Data were entered using a double-entry method. Missingness was evaluated using Little’s MCAR test. Cases with > 10% missing in critical variables were excluded. Nursing staff received structured training prior to PMNC protocol implementation to standardize delivery. Adherence data were monitored weekly by a quality assurance nurse manager to ensure fidelity and reduce intervention variability.

## Results

### Baseline characteristics of the study population

A total of 235 patients who underwent laparoscopic radical prostatectomy were included in the final analysis, with 120 patients in the PMNC group and 115 in the control group. As shown in Table 1, the two groups were generally well balanced in terms of baseline demographic and clinical characteristics. The mean age was significantly lower in the PMNC group compared to the control group (65.6 ± 4.6 vs. 68.2 ± 4.7 years, *P* < 0.001). Body mass index, ASA physical status distribution, smoking history, hypertension, and diabetes mellitus showed no statistically significant differences between the two groups (*P* > 0.05 for all). No significant differences were observed in oncologic parameters including preoperative PSA levels, Gleason scores, and clinical T stage distributions. These findings indicate that the tumor burden and disease severity at baseline were comparable across groups. There were no statistically significant differences between groups in the proportion of nerve-sparing procedures (PMNC 46.7% vs. control 44.3%), vesicourethral anastomosis method (continuous suture: PMNC 78.3% vs. control 80.0%), or extent of pelvic lymph node dissection (extended dissection: PMNC 22.5% vs. control 20.9%; all *P* > 0.05). However, surgical duration was significantly shorter in the PMNC group (158.6 ± 19.3 min vs. 169.4 ± 20.1 min, *P* < 0.001), and estimated intraoperative blood loss was also lower (202.5 ± 75.6 mL vs. 222.7 ± 81.3 mL, *P* = 0.041), which may reflect intraoperative efficiency; however, because operative duration and estimated blood loss can also improve over time due to increased surgical experience, both variables were included as covariates in the multivariable analyses to account for potential temporal bias.

**TABLE 1:** Baseline characteristics of patients undergoing laparoscopic radical prostatectomy in the PMNC and control groups.

Variable	PMNC group *(n* = 120)	Control group (*n* = 115)	*P*-value
Age (years), mean ± SD	65.6 ± 4.6	68.2 ± 4.7	< 0.001
Body mass index (kg/m^2^), mean ± SD	24.1 ± 2.0	24.6 ± 2.0	0.090
ASA physical status, n (%)	0.643		
I	29 (24.2%)	22 (19.1%)	
II	67 (55.8%)	69 (60.0%)	
III	24 (20.0%)	24 (20.9%)	
Smoking history, n (%)	41 (34.2%)	39 (33.9%)	0.962
Hypertension, n (%)	52 (43.3%)	57 (49.6%)	0.351
Diabetes mellitus, n (%)	19 (15.8%)	23 (20.0%)	0.426
Preoperative PSA (ng/mL), median (IQR)	8.7 (6.0–12.3)	9.2 (6.5–13.1)	0.278
Prostate volume (mL), mean ± SD	43.8 ± 11.5	44.5 ± 12.1	0.621
Gleason score, n (%)	0.994		
≤ 6	28 (23.3%)	26 (22.6%)	
7	67 (55.8%)	65 (56.5%)	
≥ 8	25 (20.9%)	24 (20.9%)	
Clinical T stage, n (%)	0.923		
T1	34 (28.3%)	31 (27.0%)	
T2	60 (50.0%)	57 (49.6%)	
T3	26 (21.7%)	27 (23.5%)	
Surgical duration (minutes), mean ± SD	158.6 ± 19.3	169.4 ± 20.1	< 0.001
Estimated blood loss (mL), mean ± SD	202.5 ± 75.6	222.7 ± 81.3	0.041

PMNC, Perioperative Multidisciplinary Nursing Collaboration; ASA, American Society of Anesthesiologists; PSA, Prostate-Specific Antigen; IQR, Interquartile Range; SD, Standard Deviation. *P*-values were calculated using independent-samples *t*-test or Mann–Whitney U test for continuous variables and chi-square test for categorical variables. Bolded *P*-values indicate statistical significance (*P* < 0.05).

### Perioperative recovery and clinical outcomes

As presented in Table 2, patients in the PMNC group demonstrated significantly improved perioperative recovery profiles compared to the control group. The time to first ambulation was notably shorter in the PMNC group (mean: 14.1 h vs. 17.8 h, *P* < 0.001), and urinary catheter removal occurred earlier (mean: 4.4 days vs. 5.4 days, *P* < 0.001). Furthermore, the incidence of urinary incontinence at discharge was substantially lower in the PMNC group (32.5% vs. 56.5%, *P* < 0.001), and the mean postoperative hospital stay was significantly reduced (5.2 vs. 6.1 days, *P* < 0.001). Pain control outcomes also favored the PMNC group, with significantly lower VAS scores observed at both 24 and 48 h postoperatively (*P* < 0.001 for both comparisons). Additionally, the median time to oral intake resumption was earlier in the PMNC group (12 h vs. 16 h, *P* = 0.002), indicating enhanced functional recovery. Although the rates of postoperative complications and 30-day readmission were lower in the PMNC group, the differences did not reach statistical significance (*P* = 0.077 and *P* = 0.184, respectively). These findings collectively suggest that implementation of a multidisciplinary nursing collaboration protocol contributes to more efficient recovery and improved short-term clinical outcomes following laparoscopic radical prostatectomy.

**TABLE 2:** Comparison of perioperative recovery and clinical outcomes between the PMNC and control groups.

Variable	PMNC group (*n* = 120)	Control group (*n* = 115)	*P*-value
Time to first ambulation (hours), mean ± SD	14.1 ± 3.4	17.8 ± 3.4	**< 0.001**
Time to catheter removal (days), mean ± SD	4.4 ± 0.9	5.4 ± 1.1	**< 0.001**
Urinary incontinence at discharge, n (%)	39 (32.5%)	65 (56.5%)	**< 0.001**
Length of postoperative stay (days), mean ± SD	5.2 ± 1.1	6.1 ± 1.2	**< 0.001**
Time to oral intake (hours), median (IQR)	12 (10–16)	16 (12–20)	**0.002**
Pain score at 24 h (VAS), mean ± SD	3.2 ± 0.9	4.2 ± 0.9	**< 0.001**
Pain score at 48 h (VAS), mean ± SD	2.0 ± 0.8	3.1 ± 1.0	**< 0.001**
Postoperative complications, n (%)	10 (8.3%)	18 (15.7%)	0.077
Fever ≥ 38.5°C, n (%)	4 (3.3%)	7 (6.1%)	–
Urinary tract infection, n (%)	3 (2.5%)	6 (5.2%)	–
Wound infection, n (%)	2 (1.7%)	3 (2.6%)	–
30-day readmission, n (%)	6 (5.0%)	11 (9.6%)	0.184

PMNC, Perioperative Multidisciplinary Nursing Collaboration; SD, Standard Deviation; IQR, Interquartile Range; VAS, Visual Analog Scale (0–10 pain score). *P*-values were calculated using independent-samples *t*-tests or Mann–Whitney U tests for continuous variables, and chi-square tests for categorical variables. Bolded *P*-values indicate statistical significance at the level of *P* < 0.05. Subtypes of postoperative complications are presented descriptively and were not individually analyzed due to limited event counts.

### Multivariate analysis of urinary incontinence at discharge

To identify independent predictors of urinary incontinence at discharge following laparoscopic radical prostatectomy, a multivariate logistic regression analysis was performed, with results summarized in Table 3. After adjusting for age, body mass index (BMI), ASA physical status, and surgical duration, perioperative multidisciplinary nursing collaboration (PMNC) remained independently associated with lower odds of urinary incontinence at discharge. Specifically, patients in the PMNC group had a 53% lower odds of incontinence compared with those in the control group (adjusted OR: 0.47; 95% CI: 0.28–0.80; *P* = 0.005). Although the PMNC group was significantly younger at baseline, age was included as a covariate in the regression model and was not independently associated with postoperative incontinence (*P* > 0.05), suggesting that the observed effect of PMNC on continence recovery was not explained by age differences between groups. These findings suggest that the implementation of PMNC protocols plays a decisive and independent role in improving early urinary continence recovery, beyond the influence of baseline patient characteristics and surgical factors.

**TABLE 3:** Multivariate logistic regression analysis for predictors of urinary incontinence at discharge.

Predictor	Adjusted OR (95% CI)	*P*-value
PMNC group (vs. control)	0.47 (0.28–0.80)	**0.005**
Age (per year increase)	1.02 (0.97–1.08)	0.386
BMI (kg/m^2^)	1.01 (0.88–1.16)	0.892
ASA Class (per unit increase)	1.14 (0.75–1.73)	0.539

PMNC, Perioperative Multidisciplinary Nursing Collaboration; OR, Odds Ratio; CI, Confidence Interval; BMI, Body Mass Index; ASA, American Society of Anesthesiologists. Adjusted odds ratios were estimated using multivariate logistic regression analysis. The final model was adjusted for group assignment, age, BMI, and ASA class; surgical duration was initially included but removed due to lack of statistical significance. Bolded *P*-values indicate statistical significance (*P* < 0.05). The model demonstrated acceptable explanatory power (Pseudo *R*^2^ = 0.12).

### Adherence to PMNC protocol components

Adherence to the predefined components of the perioperative multidisciplinary nursing collaboration (PMNC) protocol was generally high among patients in the intervention group. As shown in Table 4, preoperative education was completed in 95.0% of cases, and psychological support was documented in over 90%. Key intraoperative measures—including standardized SBAR handover and positioning checklist completion—were successfully implemented in 90.0 and 86.7% of cases, respectively.

**TABLE 4:** Adherence to perioperative multidisciplinary nursing collaboration (PMNC) protocol components in the intervention group (*n* = 120).

Phase	Protocol component	n adherent (%)
Preoperative	Preoperative education completed	114 (95.0%)
Psychological support documented	109 (90.8%)	
Intraoperative	SBAR handover documentation completed	108 (90.0%)
Intraoperative positioning checklist completed	104 (86.7%)	
Temperature and fluid warming monitored	105 (87.5%)	
Postoperative	Early mobilization within 24 h achieved	105 (87.5%)
Pain assessment completed (24 h)	115 (95.8%)	
Pain assessment completed (48 h)	113 (94.2%)	
Multidisciplinary nursing round conducted (Post-op Day 1)	109 (90.8%)	

PMNC, Perioperative Multidisciplinary Nursing Collaboration; SBAR, Situation, Background, Assessment, Recommendation. All items reflect predefined quality indicators from the PMNC protocol. Values represent the number and percentage of patients in the intervention group for whom each item was completed and documented. No structured implementation of these components was conduct.

Postoperative adherence was similarly strong. Early mobilization within 24 h was achieved in 87.5% of patients, and pain assessments were completed for over 94% of patients at both 24 and 48 h postoperatively. Multidisciplinary nursing rounds were conducted on postoperative day 1 in 90.8% of patients. These results confirm that the PMNC intervention was implemented with a high level of fidelity, supporting the internal validity of the observed improvements in perioperative outcomes.

### Subgroup analysis of risk factors for urinary incontinence

To explore potential risk factors for urinary incontinence at discharge among patients who did not receive PMNC intervention, a stratified analysis was performed within the control group (Table 5). Although none of the examined variables reached statistical significance, several trends were observed. Patients with ASA class III exhibited a higher incontinence rate (58.3%) compared to those with ASA I–II (47.3%), and similar patterns were noted in those with limited preoperative mobility or lacking intraoperative warming documentation. Patients aged ≥ 70 years and those with BMI ≥ 25 kg/m^2^ showed no meaningful difference in incontinence incidence compared to their counterparts. These findings suggest that perioperative physiological vulnerability and the absence of consistent intraoperative nursing practices may contribute to worse continence outcomes, reinforcing the value of comprehensive and standardized nursing protocols in high-risk subgroups.

**TABLE 5:** Urinary incontinence rates by risk factors in the control group (*n* = 115).

Risk factor	n (%)	Incontinence rate (%)	*P*-value
Age ≥ 70 vs. < 70	36 (31.3%)	52.8% vs. 48.7%	0.890
BMI ≥ 25 vs. < 25 (kg/m^2^)	48 (41.7%)	50.0% vs. 49.0%	1.000
ASA III vs. I–II	24 (20.9%)	58.3% vs. 47.3%	0.522
Preop walking limitation	15 (13.0%)	60.0% vs. 48.1%	0.381
No intraop warming record	12 (10.4%)	66.7% vs. 47.6%	0.191

ASA, American Society of Anesthesiologists; BMI, Body Mass Index. Urinary incontinence was assessed at hospital discharge. *P*-values were calculated using chi-square tests. The overall incontinence rate in the control group was 50.0%. Although none of the subgroup comparisons reached statistical significance (*P* ≥ 0.05), a trend toward higher incontinence was observed in patients with ASA class III, preoperative walking limitation, or absence of intraoperative warming documentation.

## Discussion

This retrospective cohort study demonstrated that implementation of a perioperative multidisciplinary nursing collaboration (PMNC) protocol was associated with significantly improved recovery outcomes in patients undergoing laparoscopic radical prostatectomy (LRP). Specifically, patients in the PMNC group exhibited earlier ambulation, reduced time to catheter removal, lower incidence of urinary incontinence at discharge, shorter hospital stay, and lower postoperative pain scores compared to those receiving conventional care. Importantly, multivariate analysis confirmed PMNC as an independent protective factor for early continence recovery. These findings highlight the value of structured, nurse-led perioperative care models in optimizing functional outcomes after urologic surgery.

These results are consistent with and extend previous findings from ERAS-focused studies in urologic and colorectal surgery. For example, Lin et al. (13) demonstrated that an ERAS protocol for prostate cancer patients undergoing LRP shortened hospital stay and facilitated earlier catheter removal, while Zhao et al. (14) confirmed in a meta-analysis that ERAS improved pain control and continence recovery following radical prostatectomy. Similarly, You et al. (15) reported that multidisciplinary ERAS nursing interventions improved mobilization and reduced complications in colorectal cancer patients. By aligning with and expanding upon these studies, our findings provide novel evidence that a structured, nursing-led multidisciplinary protocol (PMNC) is both feasible and effective in the context of prostate cancer surgery.

Early mobilization and timely removal of urinary catheters are critical indicators of functional recovery following LRP. Previous studies have identified these parameters as predictors of decreased risk of postoperative complications and improved patient satisfaction (16, 17). In our study, the mean time to first ambulation was 3.7 h earlier in the PMNC group, a difference that may reflect enhanced preoperative education, pain management, and mobilization support. Similarly, early catheter removal, observed nearly 1 day sooner in the PMNC group, aligns with recommendations from Enhanced Recovery After Surgery (ERAS) guidelines, which emphasize the importance of minimizing catheter dwell time to reduce urinary tract infections and improve continence recovery (18, 19). Although the average postoperative hospital stay in our study (approximately 5 days) was longer than that reported in some Western ERAS cohorts, this reflects regional practice patterns and institutional discharge criteria in China. In our center, patients are typically discharged only after urinary catheter removal, independent voiding confirmation, and completion of initial pelvic floor training under nursing supervision. Additionally, cultural expectations and medical insurance policies encourage patients to remain hospitalized until functional recovery is deemed stable. Within this context, the 1-day reduction in hospital stay observed under the PMNC protocol represents a meaningful improvement in perioperative efficiency while maintaining patient safety and satisfaction. The observed reduction in operative duration and intraoperative blood loss in the PMNC group may, in part, reflect the cumulative experience and procedural refinement of the surgical and anesthetic teams over time. Although the same core surgeons and nursing staff performed all procedures using an unchanged laparoscopic technique, gradual improvement in efficiency and teamwork is expected in longitudinal single-center studies. To minimize potential bias from temporal effects, we conducted sensitivity analyses including operative year, duration, and blood loss as covariates, which did not alter the direction or significance of the main findings. Nevertheless, the possibility that enhanced team proficiency contributed to better recovery outcomes cannot be completely excluded and was acknowledged as a contextual factor.

Urinary incontinence remains one of the most distressing postoperative complications for patients undergoing prostatectomy and has a profound impact on quality of life (20). In the present study, the rate of incontinence at discharge was significantly lower in the PMNC group (32.5% vs. 56.5%). This improvement may be attributed to standardized interventions such as early ambulation, intraoperative thermoregulation, and consistent nursing-led continence education. These elements have previously been linked to improved pelvic floor muscle function and bladder control postoperatively (21, 22). Moreover, subgroup analysis in the control group suggested that patients with higher ASA status or limited preoperative mobility were more vulnerable to continence delay, supporting the need for tailored multidisciplinary interventions in high-risk populations (23).

Pain control represents another key component of recovery. Our findings showed significantly lower visual analog scale (VAS) scores at both 24 and 48 h in the PMNC group. Effective pain management not only facilitates early mobilization but also reduces sympathetic stress responses and inflammatory cascades that can impair wound healing and bladder function (24, 25). Previous studies have demonstrated that structured nurse-driven pain assessment and timely analgesia administration are crucial in the success of ERAS and similar recovery protocols (26, 27).

The high adherence rates (≥ 85%) across all PMNC protocol components in our study suggest robust implementation fidelity, which is essential for translating evidence-based interventions into measurable clinical benefits. This consistency reinforces the feasibility and scalability of the PMNC model in routine urologic practice. In contrast to physician-led protocols, the nurse-led nature of PMNC may offer enhanced continuity of care and real-time responsiveness to patient needs, as supported by literature in colorectal and gynecologic surgery domains (28, 29).

Several mechanisms may underlie the observed improvements in recovery. First, the SBAR (Situation, Background, Assessment, Recommendation) handover technique likely enhanced interdepartmental communication and care coordination, reducing perioperative errors and delays (30). Second, structured psychological support may have mitigated preoperative anxiety, which has been independently associated with increased postoperative pain and delayed recovery in prostate cancer patients (31). Third, intraoperative thermoregulation and fluid management, often neglected in routine care, were closely monitored under PMNC, potentially reducing metabolic stress and facilitating hemostasis (32). Intraoperative thermoregulation, by maintaining core body temperature ≥ 36°C, may have reduced shivering, coagulopathy, and tissue hypoperfusion, thereby supporting hemostasis and minimizing postoperative fatigue—factors that facilitate earlier mobilization and pelvic floor training, ultimately contributing to improved continence recovery. Similarly, standardized pain assessments at 24 and 48 h ensured timely analgesic adjustments, which not only enhanced patient comfort but also removed a major barrier to ambulation and functional rehabilitation. These targeted interventions likely acted synergistically with other PMNC components to shorten time to ambulation, reduce catheter dwell time, and decrease length of stay.

This study has several limitations that should be acknowledged. First, the retrospective, single-center design and non-randomized group allocation introduce the possibility of selection and temporal bias, even though multivariable adjustment and sensitivity analyses were applied. Second, the main methods relied on retrospective extraction of outcomes from electronic medical and nursing records; therefore, patient-reported quality-of-life outcomes and long-term continence recovery beyond discharge could not be evaluated. Third, the PMNC protocol was implemented as a composite bundle of eight elements, making it impossible to disentangle the relative contribution of each component. Fourth, adherence was assessed through nursing documentation audits, which, while standardized, may still be subject to reporting bias. Finally, we did not conduct a cost-effectiveness analysis, which is essential for evaluating scalability in resource-limited settings. Future prospective, multicenter studies with longer follow-up and patient-centered endpoints are warranted to validate and extend these findings.

## Conclusion

In conclusion, this retrospective study found that implementation of a standardized, nursing-led perioperative multidisciplinary nursing collaboration (PMNC) protocol was associated with significantly better early recovery outcomes after laparoscopic radical prostatectomy, including earlier ambulation, more timely urinary catheter removal, lower urinary incontinence rates at discharge, shorter hospital stay, and reduced postoperative pain. These findings suggest that the PMNC model may provide a pragmatic and potentially scalable nursing-driven approach to support enhanced recovery in urologic oncology. Future multicenter, prospective studies with long-term follow-up and health-economic evaluations are warranted to validate these findings and further define the role of multidisciplinary nursing pathways in prostate cancer surgery.

## Data Availability

The original contributions presented in this study are included in this article/Supplementary material, further inquiries can be directed to the corresponding author.
