# Supplementary material for: Nursing-led multidisciplinary ERAS collaboration improves early recovery after laparoscopic radical prostatectomy for localized prostate cancer: a retrospective cohort study
Source: Front Med (Lausanne). 2026 Jan 14;12:1705709. doi: 10.3389/fmed.2025.1705709 (PMC12847320; doi:10.3389/fmed.2025.1705709)
Supplement: Supplementary file 1 [file Table_1.docx]

**Supplementary Table 1.** Comparison of Perioperative Interventions Between the PMNC and Standard-Care Groups

| **Phase** | **PMNC Group (Nursing-Led Multidisciplinary ERAS Collaboration)** | **Standard-Care Group (Conventional Perioperative Care)** |
| --- | --- | --- |
| **Preoperative Education** | Structured 15–20 min session conducted by trained perioperative nurses using illustrated handouts; content covered surgical steps, postoperative goals, pelvic floor muscle training, pain expectations, and catheter care; patient understanding verified via teach-back method. | Brief verbal instructions on fasting and admission logistics; no standardized materials or comprehension check. |
| **Psychological Support** | Screening with Hospital Anxiety and Depression Scale (HADS) one day before surgery; individualized counseling and relaxation-breathing guidance. | No routine psychological screening or counseling unless requested by physician. |
| **Ward–OR Communication** | Standardized **SBAR (Situation–Background–Assessment–Recommendation)** handover tool between ward and OR nurses ensuring complete transfer of clinical information and perioperative plans. | Informal verbal handover without standardized structure or documentation. |
| **Intraoperative Positioning** | Dual-nurse positioning checklist completed before incision; protection of pressure points and fixation of catheters/drains verified; documentation required. | Positioning performed per surgeon’s preference; no formal checklist or documentation. |
| **Thermoregulation and Fluid Monitoring** | Active warming devices used to maintain core temperature ≥ 36 °C; fluid balance monitored hourly and recorded. | Passive warming as needed; no target temperature or hourly fluid documentation. |
| **Early Mobilization** | Assisted sitting within 8 h and walking ≥ 10 m within 24 h post-op unless contraindicated; progress documented by nursing staff. | Mobilization at nurse’s discretion, typically after postoperative day 1; no quantitative target or record. |
| **Pain Assessment and Analgesia** | Pain assessed using 0–10 VAS at 24 h and 48 h; if VAS ≥ 4, analgesia adjusted and reassessed within 1 h; standardized nursing documentation. | Pain assessment on patient request only; analgesia adjusted irregularly; no fixed reassessment protocol. |
| **Multidisciplinary Rounds** | Structured round on postoperative day 1 involving ward nurse, charge nurse, and urology resident reviewing pain, mobility, continence, wound status, and discharge readiness. | Routine surgeon-led ward round; nursing input unsystematic; no multidisciplinary format. |

**Abbreviations:** PMNC = Perioperative Multidisciplinary Nursing Collaboration; ERAS = Enhanced Recovery After Surgery; SBAR = Situation–Background–Assessment–Recommendation; VAS = Visual Analogue Scale.
